# Genetic Variants Involved in One-Carbon Metabolism: Polymorphism Frequencies and Differences in Homocysteine Concentrations in the Folic Acid Fortification Era

**DOI:** 10.3390/nu9060539

**Published:** 2017-05-25

**Authors:** Josiane Steluti, Aline M. Carvalho, Antonio A. F. Carioca, Andreia Miranda, Gilka J. F. Gattás, Regina M. Fisberg, Dirce M. Marchioni

**Affiliations:** 1 Department of Nutrition, School of Public Health, Sao Paulo University, Avenida Doutor Arnaldo, 715-Cerqueira César, São Paulo—SP, São Paulo 01246-904, Brazil; aline.carvalho.usp@gmail.com (A.M.C.); nutriaugusto@gmail.com (A.A.F.C.); andreia.am.miranda@gmail.com (A.M.); rfisberg@usp.br (R.M.F.); marchioni@usp.br (D.M.M.); 2 Department of Legal Medicine, Bioethics and Occupational Health, School of Medicine, University of São Paulo, Avenida Doutor Arnaldo, 455-Cerqueira César, São Paulo—SP, São Paulo 01246-903, Brazil; gfgattas@usp.br

**Keywords:** one-carbon metabolism, folic acid fortification, genetic variants, homocysteine, polymorphisms

## Abstract

Folate and other B vitamins are essential co-factors of one-carbon metabolism, and genetic variants, such as polymorphisms, can alter the metabolism. Furthermore, the adoption of food fortification with folic acid showed a decrease of homocysteine concentration. The aim of this study was to investigate the frequencies of the polymorphisms of enzymes and carrier proteins involved in one-carbon metabolism, and to evaluate homocysteine concentrations in the presence of these genetic variants in a population exposed to mandatory food fortification with folic acid. Using data from a population-based cross-sectional study in São Paulo, Brazil, the study population comprised 750 participants above 12 years of age of both genders. A linear regression model was used to evaluate the homocysteine concentrations according to genetic variants and folate level. The results showed that the minor allelic frequencies were 0.33 for MTHFR (rs1801133), 0.24 for MTHFR (rs1801131), 0.19 for MTR (rs1805087), 0.42 for MTRR (rs1801394), 0.46 for RFC1 (rs1051266), and 0.47 for DHFR (19-bp deletion). The genetic variants of MTHFR 677C>T, MTRR 66A>G and RFC-1 80G>A were different according to race. The homocysteine concentrations increased in the CT and TT compared to CC genotypes of polymorphism *MTHFR* 677C>T in all populations, and differences between the homocysteine concentrations according to the genotypes of *MTHFR* 677C>T were observed regardless of folate level.

## 1. Introduction

**Figure 1:**
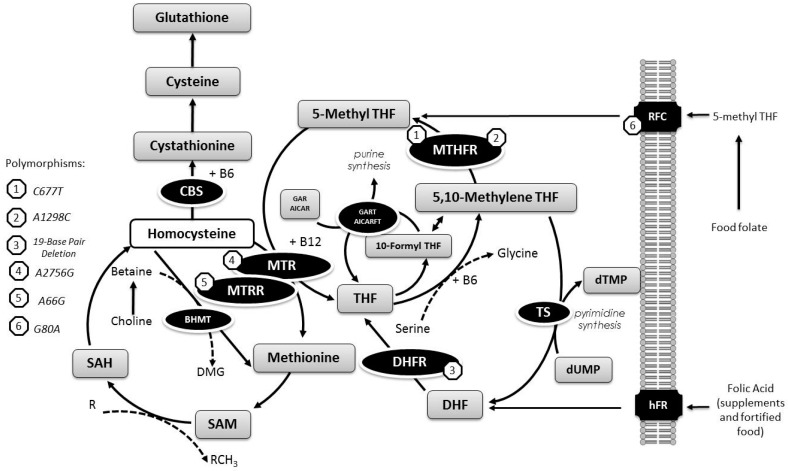
Overview of one-carbon metabolism considering metabolites, enzymes, coenzymes, and genetic variants. Abbreviations: methylenetetrahydrofolate reductase (MTHFR), methionine synthase (MTR), methionine synthase reductase (MTRR), reduced folate carrier (RFC), dihydrofolate reductase (DHFR), tetrahydrofolate (THF), cystathionine β-synthase (CBS), dihydrofolate (DHF), human folate receptor (hFR), *S*-adenosylmethionine (SAM), *S*-adenosylhomocysteine (SAH), thymidylate synthase (TS), deoxyuridine monophosphate (dUMP), deoxythymidine monophosphate (dTMP), Betaine--Homocysteine *S*-Methyltransferase (BHMT), dimethyglycine (DMG), glycinamide ribonucleotide (GAR), 5-aminoimidazole-4-carboxamide ribonucleotide (AICAR), glycinamide ribonucleotide transformylase (GART), 5-aminoimidazole-4-carboxamide ribonucleotide formyltransferase (AICARFT).

One-carbon metabolism plays an important role in complex and essential metabolic pathways, as hundreds of intracellular transmethylation reactions, including DNA methylation and DNA synthesis, have been implicated in carcinogenesis [1] and processes closely associated with homocysteine (Hcy) metabolism [2]. Vitamins, particularly folate (B9) and other B vitamins, such as B6 and B12, are essential co-factors of one-carbon metabolism (Figure 1) [3]. 

Studies have shown that the elevation of homocysteine levels is an important risk marker for the occurrence of adverse events, such as dementia, Alzheimer’s disease, bone fractures, cancers, and particularly cardiovascular diseases [4,5]. Environmental and genetic factors modify homocysteine concentrations, for example, polymorphisms can alter metabolism, generating homocysteine accumulation [6]. However, increases in the levels of some vitamins, particularly folate and other vitamins, such as B6 and B12, modulates metabolic functions [5], consequently facilitating the remethylation of homocysteine, thereby hindering increases in the concentrations of this metabolite [6]. As a result, an important reduction of homocysteine concentration in some populations was observed after the adoption of food fortification with folic acid (synthetic form of folate) in several countries [7,8].

This observation highlights the importance of adequate nutrient intake, knowledge concerning genetic variant frequencies, and evaluation of country conditions in which the mandatory policy is to fortify foods with folic acid, as has occurred in Brazil since 2002. Furthermore, despite the accumulated evidence, there are gaps in the knowledge of the frequencies of genetic variants in several primarily healthy populations, and the impact of individual responses to diseases remains unknown. The objective of the present study was to investigate the frequency of the genetic variants involved in one-carbon metabolism and to evaluate homocysteine concentrations in accordance with the presence of these genetic variants considering the folate plasma level. 

## 2. Materials and Methods 

### 2.1. Study and Sample Population

We used the data from ‘Health Survey of Sao Paulo’ (ISA-Capital), a population-based and cross-sectional household health survey conducted in 2008 [9]. The study population comprised residents of private households in the urban area of São Paulo, Brazil. This complex probabilistic sample was obtained using conglomerates in two stages: census and household sectors (data from National Survey of Households 2005, IBGE). Six sample domains, comprising adolescents (12 to 19 years of age), adults (20 to 59 years of age), and elderly (60 years of age and older) individuals of both genders, were considered, and a total of 2691 participants were included to facilitate general data collection. Among these participants, blood samples and blood pressure and anthropometric measurements were obtained from 750 individuals. Other details of the sampling have been previously published [10].

In the present study, we considered all participants aged 12 years or older at the time of collection. A total of 750 individuals (158 adolescents, 303 adults, and 289 elderly) responded to a social-economic survey and underwent anthropometric measurement, and subsequently, blood samples were collected for genotyping. 

The Ethical and Research Committee of the School of Public Health University of São Paulo (Protocol number 2001) approved this study, and informed consent was obtained from all participants.

### 2.2. Data Collection and Processing

In 2008, household information was obtained from randomly selected residents using structured questionnaires applied by previously trained interviewers. Demographic, social-economic, lifestyle, referred morbidity, family-history diseases, supplementation, medicine, and diet information were collected. In a subsequent home visit, blood samples were collected and anthropometric measurements were recorded.

### 2.3. Diet

Two 24-h recalls (24hR) were performed as a dietary survey. Food consumption described in both recalls was converted into energy and nutrient values using the Nutrition Data System for Research (NDSR, version 2007, Nutrition Coordinating Center (NCC), University of Minnesota, Minneapolis, MN, USA). This software is used to calculate the folate quantity in three different ways: (1) natural folate—the vitamin naturally present in food; (2) synthetic folate (folic acid)—vitamin added to fortified food and dietary supplements; and (3) dietary folate equivalents (DFE)—sum of the dietary quantity of natural folate and synthetic folate, considering the difference in the bioavailability of both forms. Additionally, synthetic folate values and, consequently, the DFE values were corrected, considering the mandatory fortification of wheat and corn flour in Brazil since 2004. Moreover, the Multiple Source Method (MSM) was used to measure folate intake to estimate the usual consumption distribution of nutrients, mitigating the effects of intra-individual variation when at least two dietary measurements per individual are available [11]. 

### 2.4. Anthropometric Measures

Weight and height were measured and the anthropometric data were used to calculate Body Mass Index (BMI), and the individuals were classified, according to the BMI cut-off points of the World Health Organization (WHO) [12], as underweight—BMI < 18.5 kg/m^2^; eutrophic—BMI 18.5–24.9 kg/m^2^, and overweight—BMI ≥ 25 kg/m^2^. A previously trained nurse technician performed all measurements. 

### 2.5. Blood Collection

A trained nurse technician performed blood collection at home using disposable needles and syringes according to the standardized procedures described in the Guidelines for the Assessment of Food Consumption in population studies which reported an experience of the Health Survey in São Paulo [9]. The blood samples were collected through venipuncture after 12-h fasting.

Approximately 20 mL of blood were collected in tubes containing EDTA (ethylenediaminetetraacetic acid) and plastic serum tubes which had spray-coated silica through venipuncture. The tubes were stored in Styrofoam packages with recyclable ice packs and were transported to the Laboratory of Human Nutrition at School of Public Health, followed by centrifugation and processing into aliquots of serum and plasma, and storage at −80 °C.

### 2.6. Biochemical Analysis

The folate dosage was determined through High-Performance Liquid Chromatography (HPLC) with electrochemical detection [13]. The B6 concentrations were analyzed by HPLC with fluorometric detection using the ImmunDiagnostik AG^®^ HPLC-Analytik system [14]. Vitamin B12 concentrations were determined by a chemiluminescence immunoassay with paramagnetic particles for the quantitative determination of vitamin levels in human serum and plasma using the Access Immunoassay System^®^ (Beckman Coulter, Inc., Galway, Ireland). The immunoassay method of chemiluminescence microparticles using the ARCHITECT Homocysteine Reagent Kit (Abbott Diagnostics Division, Abbott Park, IL, USA) was used to analyze the plasma concentrations of homocysteine. All tests were performed according to the manufacturer’s instructions. 

Mean intra-assay coefficients of variation (CVs) for B6, B12, folate, and homocysteine, respectively, were 7.2%, 7.0%, 2.0%, and 2.3%, and mean inter-assay CVs were 5.9%, 8.5%, 3.4%, and 4.0%, respectively. 

### 2.7. DNA Extraction and Genotyping

The DNA was extracted using the DNA salt extraction method [15]. Subsequently, the DNA was quantified using a Nanodrop^®^ 1000 Spectrophotometer (Wilmington, DE, USA). 

The PCR-allele technique was used for genotyping in duplicates with 100% of concordance [16]. This assay facilitates the simultaneous amplification and detection of DNA using a common reverse primer in each reaction tube and two marked primers with two different fluorophores that recognize specific sequences corresponding to each allele. A fluorescence reader was used to capture the emitted fluorescence signal for clusterizing and identifying genotypes. 

The following six polymorphisms in the genes involved in folate metabolism were analyzed: 677C>T (rs1801133) and 1298A>C (rs1801131) of methylenetetrahydrofolate reductase—MTHFR; 2756A>G (rs1805087) of methionine synthase—MTR; 66A>G (rs1801394) of methionine synthase reductase—MTRR; 80G>A (rs1051266) of reduced folate carrier 1-RFC-1; and a 19-bp deletion in dihydrofolate reductase—DHFR. In this study, the genotyping call rate was >97% for the polymorphisms. 

### 2.8. Statistical Analysis

For each polymorphism in the population, the minimum allele frequency was calculated, and the Hardy-Weinberg equilibrium was verified. The frequencies of female and male, age group, and self-declared races, and median of folate and homocysteine concentrations were stratified according to genotypes for each of the gene encoding enzymes or carrier proteins. The chi-square test and Kruskal-Wallis test were performed to verify differences between frequencies and medians, respectively. The folate concentration was also considered to evaluate the differences in homocysteine concentrations in the presence of genetic variants. Therefore, the population was divided into tertiles of the folate plasma concentration with mean, respectively: first (14.9 nmol/L), second (27.5 nmol/L), and third (50.7 nmol/L). A linear regression model was used to evaluate differences in the homocysteine concentrations in accordance with genetic variants by total population and folate concentration tertiles. The model considered the log-transformed homocysteine concentration as a dependent variable and the genetic variants as independent variables, divided into three categories: (1) homozygous wild-type, (2) heterozygous mutation, and (3) homozygous mutation for enzyme or carrier genotypes. The models were adjusted according to race, sex, age, estimated consumption of total folate (mcg of DFE/day), body mass index, serum concentrations of vitamins, folate, B6, and B12. In addition, we used the interaction terms of genotypes and folate plasma concentration in these linear regression models. 

All statistical analyses were done using the STATA^®^ software (version 10.0, 2007; College Station, TX, USA). A significance level of 5% was considered in all analyses. 

## 3. Results

The present study involved a total of 750 participants as a representative sample of the population of São Paulo, Brazil, of which 58.1% of the participants were women and 41.9% of the participants were men, averaging 46.7 years of age (95% CI: 45.0–48.4 years). In relation to the nutritional status of the population according to the BMI classification, 46.1% of the population was overweight (BMI > 25 kg/m^2^). Moreover, in terms of race, most of the participants were self-declared Whites (59.2%), followed by Mixed (white/black) (30.7%), Blacks (8.3%), and Asians/Indigenous (1.9%). Non-smokers represented 85.2% of the population. The median of homocysteine and folate concentrations in the studied population were 8.8 μmol/L and 27.4 nmol/L, respectively. 

The primary information concerning the polymorphisms studied in this population, such as gene location, change of DNA molecule bases and consequent amino acid alterations, Hardy-Weinberg equilibrium, and minor allele frequencies, are listed in Table 1. The chi-square test (*p* > 0.05) revealed that the population was in Hardy-Weinberg equilibrium for all of the alleles studied. The genotype frequencies of the genetic variants in the studied population according to sex, age group, and self-declared race, and the respective homocysteine and folate concentrations according to genotypes are listed in Table 2. Individuals presenting genotypes CT and TT for the MTHFR 677C>T polymorphism presented higher homocysteine levels than individuals with genotype CC (*p* = 0.026), whereas individuals presenting genotypes GA and GG for the MTR 2756A>G polymorphism presented lower folate concentrations than individuals with genotype AA (*p* = 0.015). Race was observed to show statistically significant differences in the genotypes of MTHFR 677>T (*p* = 0.035), MTRR 66A>G (*p* = 0.000), and RFC-1 80G>A (*p* = 0.003).

The association between genetic variants involved in one-carbon metabolism and homocysteine concentration was assessed (Table 3). Only MTHFR 677C>T was significantly associated with Hcy concentration in the total population (*p* = 0.000). The increasing risk allele (T) was related to the increase in Hcy concentration. In the presence of genetic variants stratified by folate level tertiles, the same effect was observed in MTHFR 677C>T. The individuals carrying the risk allele had higher Hcy concentrations than those of non-carriers in the first tertile (*p* = 0.006) and third tertile (*p* = 0.038) of folate concentration. However, no interaction was observed between genotypes and folate concentration on homocysteine concentration (*p* > 0.05).

## 4. Discussion

Herein, we conducted a population-based study of individuals in the city of São Paulo, Brazil, to evaluate the frequencies of the primary polymorphisms associated with one-carbon metabolism, the associations between these genotypes and sex, age, and self-declared race, and the differences in metabolic responses considering genetic variants. The results showed that (1) the allele frequencies in the population were in equilibrium and similar to those in other populations; (2) the genotype frequencies of MTHFR 677C>T, MTRR 66A>G, and RFC-1 80G>A were significantly different according to race; and (3) statistically significant differences between the mean homocysteine concentrations according to the genotypes of MTHFR 677C>T were observed independently of folate level. 

Among the described polymorphisms of the enzyme MTHFR, the variants 677C>T and 1298A>C are the most well studied. Americans of Hispanic origin presented a higher prevalence of homozygotes TT for MTHFR 677C>T, which was found in 25% of the population. The prevalence of this genotype among white Americans was between 10% and 15%, and a lower prevalence of this genotype was observed for African and African-Americans, with 0% and 1%, respectively [4,17,18]. The results from case-control studies showed that the homozygote prevalence (TT) for this variant (677C>T) was 9.5% in control Brazilian individuals who were considered to be healthy [19], while the homozygote prevalence was 1.9% in individuals of African origin and 11.8% in individuals of Caucasian origin [20]. Herein, we observed that 11.3% of the population presented as being homozygous (TT) for the variant 677C>T, considering a homozygote prevalence of 13.2% and 1.6% among White and Black races, respectively. In a study concerning the polymorphism 1298A>C, 40% heterozygotes (AC) and 6% mutant homozygotes CC were observed in a healthy Brazilian population [21], consistent with the results of the present study (34.2% AC and 6.2% CC). In the United Kingdom, 44.8% heterozygotes AC and 9.3% mutant homozygotes CC were observed in an elderly population (*n* = 1041) [22]. 

The prevalence of 3.7% homozygotes GG and 19.7% heterozygotes AG for the MTR variant 2756A>G was observed in the control individuals from a Brazilian population [23], whereas in the present study, we observed a prevalence of 4.2% homozygotes and 29.8% heterozygotes. The homozygotes GG were detected at a frequency of 2–3% in Japanese, Chinese, Korean, and European individuals and at approximately 1–5% in Americans [24]. However, the MTRR polymorphism is extremely common. The prevalence of this genotype among the individuals examined in the present study was 44.8% heterozygotes AG and 19.6% homozygotes GG. However, in a previous study, the prevalence of the MTRR polymorphism among Brazilians was 54.3% and 17.6%, respectively [23]. 

The RFC-1 genetic variant is a mutation with high prevalence among the studied populations. Homozygotes AA showed a prevalence of 22.3% and heterozygotes AG showed a prevalence of 48.4% among a Brazilian population that attended a public health care center [25]. In contrast, in the present study, we detected a 22.5% prevalence of homozygotes AA and 47% prevalence of homozygotes AG, whereas in a British elderly population, the observed prevalence was 18.6% for AA and 50% for AG [22]. A 19-base pair deletion in the DHFR gene was also prevalent among these populations. The homozygote and heterozygote variants for this deletion accounted for 17.2% and 51.3%, respectively, of the population in the United States [26]. However, in the Brazilian population, the DHFR polymorphism has only been reported in studies of individuals with DS, presenting a frequency of genotype Del:Del in 20.9% of these individuals [27]. In the present study, the observed genotype frequency of Del:Del was 21.4%.

In relation to the homocysteine concentration, several studies have shown that an increase in folate consumption, particularly folic acid via fortification or supplementation, consequently increases folate intake and serum vitamin concentrations, and decreases homocysteine concentrations [28]. Additional studies have shown no alterations in the homocysteine concentration when folate is adequate. However, with low folate concentrations, there is a significant increase in the homocysteine concentration in individuals presenting genetic variants as methylenetetrahydrofolate reductase C677T mutation [29]. In contrast, the results of the present study showed that the mean homocysteine concentration was significantly lower (*p* < 0.05) in individuals with wild-type compared to the increasing risk allele (T) for the MTHFR 677C>T polymorphism regardless of folate concentrations tertiles. Similar results were found in other studies of folic acid supplementation. The mutation MTHFR TT was associated with lower folate concentrations and higher homocysteine concentration, and the trend of TT compared to CC was maintained even at different folic acid doses [30]. Indeed, for the remethylation of Hcy into methionine, the enzyme MTHFR is responsible for converting 5.10-methyltetrahydrofolate into 5-methyltetrahydrofolate, the circulating and physiologically active form of folate and primary methyl group donator for remethylation [3]. The results of a previous study showed that homozygotes (TT) for the MTHFR 677C>T mutation presented one-third of the expected activity for this enzyme [31]. It has been suggested that folic acid from fortified food primarily increases the intake and serum concentration of folate [32]. The synthetic form of folate, folic acid, needs to be converted into 5-methyltetrahydrofolate (5MeTHF) through the enzyme MTHFR. However, the presence of the MTHFR 677C>T polymorphism would restrict this conversion, thereby decreasing homocysteine remethylation and consequently increasing homocysteine concentrations in the blood [3,30]. On the other hand, the effect of natural sources of folate on plasma homocysteine has not been assessed in studies. It is known that common folate form in food without fortification, i.e., naturally occurring folate, is 5MeTHF after absorption, and this form does not require conversion by enzyme MTHFR in the metabolism [33]. Thus, we emphasized the need for further research studies to elucidate this gap. 

Another important finding is related to differences in genetic variations according to race. In the present study, race was differently presented (*p* < 0.05) among MTHFR 677>T, MTRR 66A>G, and RFC-1 80G>A genotypes. Some researchers criticize the use and limitations of self-declared race and consider the use of individual genetic ancestry to be the best measure of racial differences in genetic variations [34]. Nevertheless, significant differences in the prevalence of polymorphisms among self-declared races were observed, suggesting the importance of population miscegenation [35]. Therefore, the race variable must be considered in future population studies evaluating potential differences in serum concentrations of folate, homocysteine, B vitamins, and other molecules, consistent with previous studies [36,37]. 

Accordingly, the knowledge of genetic variability in epidemiological studies remains scarce in Brazil, primarily in representative samples of healthy populations. However, several studies have attempted to associate disease outcomes with genetic variability among individual populations. In the last two decades, several studies have reported that MTHFR polymorphisms, particularly 677C>T and 1298A>C, are associated with an increased risk for neural tube defects [38], cardiovascular diseases [39], schizophrenia [40], neoplasia [41,42,43], and hyperhomocysteinemia [44]. In contrast with other diseases related to folate metabolism, the MTR variant has not been associated with hyperhomocysteinemia, an increased risk of neural tube defects, or vascular diseases [4,45]. Several studies have investigated the association of the MTR variant with the development of neoplasia; however, the results are conflicting. The results of a meta-analysis of the MTRR 66A>G polymorphism showed that the genotype GG is associated with an increase in risk of carcinomas [46]. With respect to RFC-1, several studies have related the genetic variants for RFC-1 with an increased risk for neural tube defects [47]. The 19-base pair deletion in the DHFR gene has only been reported in studies concerning individuals with Down syndrome [27,48]. 

Despite these findings, a potential limitation of the present study was the use of self-declared race, as this variable reflects the influence of social-economic and cultural status, although, until recently, genetic ancestry analysis was not performed in population-based studies in the city of São Paulo. Accordingly, even self-declared race is not an adequate genetic indicator, but rather this variable could represent an exposition indicator of social factors [49]. Therefore, epidemiological studies with representative samples are important to promote these results for further discussions concerning this issue.

## 5. Conclusions

In conclusion, adequate folate levels are an important point in this discussion, as Brazil is a country that has a public health policy of mandatory folic acid fortification. Folic acid fortification considerably reduces the inadequacy of folate prevalence within levels considered to be safe in the inhabitant population of São Paulo [32]; however, the profile of genetic variability among this population remains unknown. Indeed, the presence of these polymorphisms modifies the metabolism and alters the concentrations of these metabolites, as differences in homocysteine concentrations were observed in individuals with genetic variants of MTHFR 677C>T. These effects in health and disease are inconclusive; therefore, the evaluation of such variants must be considered in future epidemiological studies. Consequently, studies concerning the frequencies and factors associated with genetic variants in the enzymes involved in metabolic processes are becoming increasingly important.

## Figures and Tables

**Table 1:** Panel of genetic variants, minimum allele frequency, and Hardy-Weinberg equilibrium of the six polymorphisms involved in one-carbon metabolism.

Polymorphisms	Location	Gene	Changes		*p* ^a^	MAF
			DNA	Amino acids		
rs1801131	1p36.3	MTHFR	A→C	Glu→Ala	0.392	0.24
rs1801133	1p36.3	MTHFR	C→T	Ala→Val	0.428	0.33
rs1805087	1q43	MTR	A→G	Asp→Gly	0.333	0.19
rs1801394	5p15.31	MTRR	A→G	-	0.154	0.42
rs1051266	21q22.3	RFC1	G→A	His→Arg	0.141	0.46
19-bp deletion	5q11.2–q13.2	DHFR	-	-	0.807	0.47

^a^ *p*-value for Hardy-Weinberg equilibrium. MAF, minor allele frequency; RFC-1, reduced folate carrier 1; DHFR, dihydrofolate reductase; MTHFR, 5,10-methylenetetrahydrofolate reductase; MTR, methionine synthase; MTRR, methionine synthase reductase.

**Table 2:** Genotype frequencies of the enzymes MTHFR, MTR, MTRR, and DHFR and the carrier protein RFC-1 according to sex, age group, and race; and the medians of homocysteine and folate concentrations according to genotypes.

SNP	MTHFR 677C>T			*p*-Value	MTHFR 1298A>C			*p*-Value	MTR 2756A>G			*p*-Value	MTRR 66A>G			*p*-Value	RFC1 80G>A			*p*-Value	DHFR Deletion			*p*-Value
**Genotypes**	**C:C**	**C:T**	**T:T**		**A:A**	**A:C**	**C:C**		**A:A**	**A:G**	**G:G**		**A:A**	**A:G**	**G:G**		**G:G**	**G:A**	**A:A**		**WT: WT**	**WT:del**	**del:del**	
**Total (%)**	46.0	42.7	11.3		58.6	34.2	6.2		66.0	29.8	4.2		35.6	44.8	19.6		30.5	47.0	22.5		28.4	50.2	21.4	
**Sex (%) ^1^**																								
male	43.4	44.3	12.3	0.462	60.6	34.5	4.9	0.376	67.8	27.7	4.6	0.548	35.0	45.2	19.8	0.961	31.6	45.1	23.4	0.682	20.1	52.9	27.0	0.455
female	47.8	41.6	10.6		57.2	35.6	7.21		64.7	31.3	3.9		36.0	44.5	19.5		29.7	48.4	21.9		22.4	48.2	29.4	
**Age group (%) ^1^**																								
12–19 years	42.0	44.0	14.0	0.662	61.9	33.6	4.5	0.593	69.5	26.6	3.9	0.139	33.3	44.4	22.2	0.193	28.5	45.7	25.8	0.763	24.4	48.1	27.6	0.801
20–59 years	48.2	41.2	10.6		56.0	36.3	7.7		69.5	26.2	4.4		36.9	47.8	15.4		32.1	45.7	22.2		19.4	52.0	28.6	
60+ years	45.8	43.7	10.6		59.6	34.8	5.7		60.5	35.3	4.2		35.5	41.8	22.7		29.9	48.9	21.1		21.9	49.5	28.6	
**Race (%) ^1^**																								
White	42.4	44.4	13.2	0.035 *	54.9	37.5	7.6	0.056	68.4	28.9	2.8	0.183	28.7	46.2	25.2	0.000 *	32.4	47.3	20.3	0.003 *	30.3	51.3	18.5	0.120
Black	59.7	38.7	1.6		69.4	27.4	3.2		65.6	29.5	4.9		54.8	38.7	6.5		29.5	57.4	13.1		16.1	51.6	32.3	
Mixed (white/black)	49.3	41.0	9.7		63.7	32.3	4.0		61.2	31.7	7.1		43.1	43.5	13.5		28.4	44.4	27.1		28.1	47.8	24.1	
Asian/Indigenous	42.9	35.7	21.4		41.7	41.7	16.7		71.4	28.6	0.0		42.9	50.0	7.1		7.7	30.8	61.5		28.6	50.0	21.4	
**Hcy, µmol/L (median) ^2^**	8.3	9.0	9.3	0.026 *	8.7	8.9	7.5	0.180	8.6	8.9	8.7	0.569	9.0	8.2	9.4	0.097	8.9	8.8	8.5	0.401	8.4	8.9	8.8	0.355
**Folate, nmol/L (median) ^2^**	27.9	27.9	24.1	0.088	26.5	28.5	22.8	0.565	28.0	25.1	21.0	0.015 *	26.6	27.0	28.1	0.423	29.0	27.4	25.4	0.396	27.5	28.0	25.6	0.935

^1^ *p*-value for the chi-square test; ^2^ *p*-value for the Kruskal-Wallis test; * A *p*-value < 0.05 was considered statistically significant.

**Table 3:** Associations between the genotypes involved in one-carbon metabolism and homocysteine concentration by total population and folate concentration tertiles.

Homocysteine ^1^	Total		Folate Concentration						*p*-Interaction ^3^
			First Tertile		Second Tertile		Third Tertile		
	Mean	SEM	Mean	SEM	Mean	SEM	Mean	SEM	
MTHFR 677C>T									
C:C	9.3	0.2	9.7	0.4	9.6	0.4	8.9	0.3	0.208
C:T	10.3	0.4	10.5	0.8	10.0	0.4	10.5	1.0	
T:T	12.6	1.3	12.6	1.3	10.2	1.7	13.9	3.9	
*p*-value ^2^	0.000 *		0.006 *		0.162		0.038 *		
MTHFR 1298A>C									
A:A	10.5	0.4	11.0	0.6	9.5	0.4	10.9	1.0	0.327
A:C	9.7	0.2	10.3	0.5	10.0	0.5	9.0	0.3	
C:C	9.2	0.7	7.1	0.6	12.1	1.6	8.4	1.3	
*p*-value ^2^	0.304		0.121		0.190		0.187		
MTR 2756A>G									
A:A	10.0	0.3	10.1	0.4	9.5	0.3	10.4	0.8	0.397
A:G	10.4	0.5	11.5	1.2	10.5	0.7	9.2	0.4	
G:G	9.1	0.5	9.6	0.7	8.5	1.0	9.8	1.5	
*p*-value ^2^	0.439		0.583		0.859		0.374		
MTRR 66A>G									
A:A	10.2	0.4	10.4	0.5	10.0	0.6	10.6	1.1	0.825
A:G	10.0	0.4	10.6	0.8	9.5	0.5	9.6	0.9	
G:G	10.1	0.4	9.8	0.9	10.1	0.6	10.4	0.7	
*p*-value ^2^	0.576		0.59		0.612		0.388		
RFC1 80G>A									
G:G	10.7	0.5	11.7	1.2	11.0	0.8	9.2	0.5	0.214
G:A	10.1	0.4	9.6	0.4	9.7	0.4	11.2	1.2	
A:A	9.4	0.3	10.5	0.7	8.5	0.5	9.2	0.5	
*p*-value ^2^	0.281		0.920		0.001 *		0.396		
deletion DHFR									
WT:WT	10.4	0.6	10.5	1.3	9.7	0.8	10.8	1.3	0.535
WT:del	10.1	0.3	10.2	0.4	9.8	0.4	10.1	0.8	
del:del	10.1	0.4	11.2	0.8	10.3	0.6	8.9	0.5	
*p*-value ^2^	0.312		0.071		0.667		0.836		

^1^ Hcy concentration data are mean and SEM. Hcy concentration values were Log transformed before analysis. ^2^ Linear regression model adjusted by race, sex, age, estimated consumption of total folate (mcg of DFE/day), body mass index, serum concentrations of vitamins folate, B6, and B12. ^3^ *p*-value of genotypes-folate concentration interaction term at the linear regression model. * *p*-values were considered significant (*p* < 0.05).
